# Clinical and microbiological characteristics of *Klebsiella pneumoniae* liver abscess in East China

**DOI:** 10.1186/s12879-015-0899-7

**Published:** 2015-03-27

**Authors:** Ting-ting Qu, Jian-cang Zhou, Yan Jiang, Ke-ren Shi, Bin Li, Ping Shen, Ze-qing Wei, Yun-song Yu

**Affiliations:** State Key Laboratory for Diagnosis and treatment of Infectious Disease, First Affiliated Hospital, College of Medicine, Zhejiang University, 3# Qingchun East Road, Hangzhou, 310016 China; Department of Infectious Diseases, Sir Run Run Shaw Hospital, College of Medicine, Zhejiang University, Hangzhou, Zhejiang China; Department of Infectious Diseases, Forth Affiliated Hospital, College of Medicine, Zhejiang University, Yiwu, Zhejiang China; Collaborative Innovation Center for Diagnosis and Treatment of Infectious Diseases, Hangzhou, Zhejiang China

**Keywords:** *Klebsiella pneumoniae*, *K. pneumoniae* liver abscess (KLA), Hypermucoviscous, *magA*, Multilocus sequence typing (MLST)

## Abstract

**Background:**

*Klebsiella pneumoniae* has been the dominant pathogen for liver abscesses in several Asian countries. Although the prevalence of *K. pneumoniae* liver abscess (KLA) in mainland China is increasing recently, the clinical and microbiological characteristics of KLA in China have not been elucidated.

**Methods:**

Clinical and microbiology characteristics of 45 consecutive patients with KLA from a tertiary teaching hospital in China between June 2008 and June 2012 were retrospectively evaluated.

**Results:**

Vast majority of the strains were susceptible to main antimicrobial agents. Most of *K. pneumoniae* strains from pyogenic liver abscess patients belonged to K1/K2 serotype (68.9% for K1 serotype and 20% for K2 serotype). All *K. pneumoniae* strains were *rmpA* positive, and 68.9% of these strains were *magA* positive. Overall, 57.8% (26/45) of *K. pneumoniae* strains belonged to ST23. Twenty-five of 26 ST23 *K. pneu*monia*e* isolates (96.2%) from KLA patients were *magA*-positive and K1 serotype. Only 28.9% (13/45) of KLA isolates exhibited hypermucoviscous phenotype, which is clinically used as the characteristic of hypervirulent *K. pneumoniae* (hvKP). Liver abscess sizes in patients infected with hvKP were tend to be larger than those in patients infected with cKP. There was no significant association between the microbiological and clinical characteristics including serotypes, *magA* and *rmpA* genotypes, and STs with the metastatic infection and prognosis of KLA.

**Conclusions:**

Neither the serotypes, *magA* and *rmpA* genotypes, nor the STs of *K. pneumoniae* were associated with the metastatic infection and prognosis of KLA. However, further studies with larger sample are needed in the future.

**Electronic supplementary material:**

The online version of this article (doi:10.1186/s12879-015-0899-7) contains supplementary material, which is available to authorized users.

## Background

*Klebsiella pneumoniae* has emerged as the dominant cause of pyogenic liver abscess in Asia and then was found worldwide. This condition is frequently associated with severe complications, including septic endophthalmitis and other extrahepatic lesions infections, especially in patients with diabetes [1-3]. This new *K. pneumoniae* variant was defined as hypervirulent *K. pneumonia* (hvKP). Furthermore, the ability for metastatic spread of infection demonstrated by hvKP is different from classic *K. pneumoniae* (cKP). Given there is no clear marker for hvKP strains, a hypermucoviscous phenotype has been considered to be associated with strains that cause KLA. This mucoid phenotype might be indicative of the extent of capsular polysaccharide expression, which is related to resistance to phagocytosis. Currently, hepato-virulent *K. pneumoniae* causing primary hepatic abscesses has posed a challenge for early laboratory identification and recognition. Bacteraemic *K. pneumoniae* isolates with positive string tests should be considered invasive strains capable of causing disseminated infection because the increased virulence associated with hyper virulent (HV) strains [4,5].

Capsular serotypes, *magA*, and *rmpA* have been documented in high prevalence for *K. pneumonia* liver abscess. Several studies of bacterial pathogenesis in Taiwan have documented that serotype K1 or K2, *magA*, and *rmpA* are possible virulence factors in *K. pneumoniae* liver abscess. The *magA* gene actually corresponds to the capsular polysaccharide synthesis (*cps*) gene *wzy* of *K. pneumoniae* isolates of serotype K1 [6-10]. The *rmpA* gene is a plasmid-mediated regulator of extracellular polysaccharide synthesis, and *rmpA*-carrying strains were associated with the hypermucoviscosity phenotype [8,11,12].

Multilocus Sequence typing (MLST) is an increasingly common used molecular epidemiologic approach for categorizing strains, and a typing screen has been described for *K. pneumoniae*. ST23 was most commonly described so far and is strongly associated with the K1 capsular serotype and liver abscess [2,13]. However, ST-65-like and −86-like are the two major MLST types among serotype K2 isolates from Asia [2,14].

In mainland China, the prevalence of *K. pneumoniae* liver abscess (KLA) is high, while only some KLA cases were reported in the English language scientific literature. Recently, Luo et al. firstly reported the molecular epidemiology and prevalence of virulence factors of the 51 KLA isolates in a Chinese hospital. However, very little clinical information was collected and evaluated in their study [15]. In our study, we analyzed clinical and microbiological characteristics of 45 KLA from a medical center in China between June 2008 and June 2012. To investigate the relationship of the clinical and microbiology characteristics of KLA, the present study evaluated the clinical manifestations of patients and detected the antimicrobial agents susceptibility, K1/K2 serotypes, MLST, hypermucoviscous phenotype, and *magA*/*rmpA* genotypes of the related *K. pneumoniae* isolates from KLA patients in a medical center over a 4-year period in East China.

## Methods

### Study design and setting

The study was a retrospective review of all patients with KLA in the First Affiliated Hospital, College of Medicine, Zhejiang University, an urban, 2500-bed major tertiary teaching hospital in Hangzhou, East China, from June 1, 2008 to June 30, 2012. The institutional review board of the First Affiliated Hospital, College of Medicine, Zhejiang University approved the study protocol and waived from the need for a consent form.

The hospital was one of the leading hospitals for liver transplantation in China and has an approximate annual admission of 108,700. It has a quaternary referral hepato-biliary unit and therefore, many patients with hepato-biliary diseases were transferred from rural hospitals to our hospital. In 2013, it became the one of the public hospitals in mainland China to be accredited by the Joint Commission International, a US-based, World Health Organization-authorized organization for medical quality evaluation.

### Data sources and definition of KLA and outcomes

The medical records were retrospectively reviewed to extract all the patients with a diagnosis of KLA during the study period. A diagnosis of a KLA was defined with the combination of presence of the typical clinical manifestations of infection, such as fever, sepsis and right upper abdominal pain, imaging evidence, and positive aspiration that was consistent with a pyogenic liver abscess (PLA). The patients with KLA who were included in our study met the following criteria: 1) a PLA was the primary cause of the hospitalization but not a complication; 2) older than 18 years old; 3) PLA patients with *K. pneumoniae* culture-positive of drained abscess. Thus, PLA patients without drainage but with positive blood cultures of *K. pneumoniae* were excluded in this study. As practiced in our hospital, lab examinations for all patients with KLA are required on the admission and generally every 2–3 days at the discretion of the attending physicians.

For each enrolled patient, the following data elements were extracted: 1) demographic characteristics (age, gender); (2) coexisting conditions; (3) location and size of abscess; (4) laboratory examinations; (5) hospital outcomes. Primary outcome was hospital mortality, and secondary outcome were complications including septic shock and metastatic infections to extra-hepatic sites, such as spontaneous bacterial peritonitis, pneumonia, endophthalmitis.

### Antimicrobial susceptibility testing

Susceptibility testing for the 45 *K. pneumoniae* strains was performed using the E-test strip according to the manufacturer’s instructions. Results were interpreted according to the recommendations and definitions from Clinical and Laboratory Standards Institute (CLSI). Antimicrobial agents tested included amikacin, amoxicillin-clavulanate, ampicillin, ampicillin-sulbactam, aztreonam, cefoxitin, cefuroxime, cefepime, cefotaxime, ceftazidime, ciprofloxacin, gentamicin, piperacillin, piperacillin-tazobactam, cefoperazone-sulbactam, tetracycline, minocycline, sulphamethoxazole-trimethoprim (SMZ-TMP), imipenem and meropenem.

### Extended-spectrum beta-lactamases (ESBLs) detection

The combination-disk synergy tests using cefotaxime (30 μg) ± clavulanic acid (10 μg) and ceftazidime (30 μg) ± clavulanic acid (10 μg) were performed to detect ESBLs phenotype for all the collected isolates. The ESBLs phenotype was confirmed by 5-mm or greatly increased zone diameter for either cefotaxime or ceftazidime in combination with clavulanic acid versus its zone when tested alone.

### String test

Strains with the hypermucoviscosity phenotype were defined as high virulent. A string test was performed to distinguish hvKP from cKP. A positive string test was defined as the formation of a viscous string >5 mm in length when bacterial colonies on an agar plate are stretched by an inoculation loop [5].

### PCR-mediated detection of *rmpA* and capsular serotype-specific genes

Genomic DNA was extracted from all *K. pneumoniae* strains (QIAGEN DNA extraction kit, QIAGEN, USA) and the *rmpA* and serotype-specific genes for the K1, and K2 capsular serotypes were amplified by polymerase chain reaction (PCR) as previously reported [16,17].

### Multilocus sequence typing (MLST) and Pulsed field gel electrophoresis (PFGE)

MLST of seven housekeeping genes (*gapA, mdh, phoE, tonB, infB, pgi, and rpoB*) was performed according to the protocol described on the *K. pneumoniae* MLST website (http://bigsdb.web.pasteur.fr/klebsiella/primers_used.html). Alleles and sequence types (STs) were assigned by using the MLST database (http://www.pasteur.fr/cgi-bin/genopole/PF8/mlstdbnet.pl?file=klebs_profiles.xml). Alleles and STs that had not been described previously were submitted to the database.

Total DNA was prepared and PFGE was performed as described previously [18]. The PFGE dendrogram was created by Software BioNumerics 6.6. The results were interpreted according to the criteria suggested by Tenoval et al. [19].

### Statistical analysis

Statistical analysis was performed using SPSS 16.0 (Chicago, Ill, USA). Descriptive data were reported as either mean ± SD or number and percentage. Normally distribution of the data was tested with Kolmogorov-Smirnov test. With respect to the differences in outcomes between various groups, categorical variables were compared using chi-square analysis. Where the number of cases was smaller than 5, the Fisher’s exact test was used. Continuous variables were compared using Independent Sample T test for normally distributed data and Mann–Whitney *U* test for non-normally distributed data. Significance was defined as a *P* value <0.05.

## Results

### Patients’ characteristics

From June 2008 to June 2012, a total of 45 liver abscess patients admitted to our hospital were diagnosed as KLA with the *K. pneumoniae* culture-positive of their liver abscess drainage. Demographic data and clinical characteristics were shown in Table 1.

**Table 1:** **Clinical characteristics of** ***K. pneumoniae*** **liver abscess in China**

**Clinical characteristic**	**Value (n, %)**
**Gender**	
Male	27(60.0%)
Female	18(40.0%)
**Age (yr)**	
mean age	53.7 ± 11.2(18–73)
18-49	14(31.1%)
50-64	23(51.1%)
≥65	8(17.8%)
**Coexisting condition**	
Diabetes mellitus	22(48.9%)
Biliary tract diseases	16(35.6%)
Abdominal surgery	10(22.2%)
Immunosuppression*	0
**No underlying diseases**	5(11.1%)
**Current drinker**	14(31.1%)
**Abscess**	
Single	40(88.9%)
Multiple	5(11.1%)
Right hepatic lobe	35(77.9%)
Left hepatic lobe	7(15.6%)
Both lobes	3(6.7%)
Abscess size (cm)	
<5	5(11.1%)
5-10	33(73.3%)
>10	7(15.6%)
**Complications**	
Metastatic infections	14(31.1%)
Spontaneous bacterial peritonitis	3(6.7%)
Pneumonia	10(22.2%)
Endophthalmitis	1(2.2%)
Septic shock	3(6.7%)
**Lab examination**	
WBC (×10^9^/L)	11.9(7.0-28.1)
CRP (mg/L)	106.9(28.8-311.0)
**Mortality**	0

*Immunosuppression may have been caused by the presence of human immunodeficiency virus infection, chemotherapy or radiotherapy within 1 month before the onset of illness, or glucocorticoid therapy (equivalent of 30 mg of prednisone per day) for 15 days before the onset of illness.

Twenty-seven (60.0%) were males and 18 (40.0%) were females. The mean age was 53.7 ± 11.2 years. Diabetes mellitus was found in 22 patients (48.9%), followed by biliary tract disease found in 16 patients (35.6%). Ten patients (22.2%) had a history of abdominal surgery. No patient was in immunosuppression condition (definition shown below Table 1), and 5 patients (11.1%) had no underlying diseases.

Forty patients had solitary abscess and 5 patients (11.1%) had multiple abscesses. Among them, 35 (77.9%) had abscesses in the right hepatic lobe, 7 (15.6%) in the left hepatic lobe, and 3 (6.7%) in both hepatic lobes. Fourteen patients (31.1%) had metastatic infections, including 3 patients with spontaneous bacterial peritonitis, 10 with pneumonia and 1 with endophthalmitis. Three patients had septic shock. Overall, none of the patients died.

Leukocytosis was found in patients with a median WBC count of 11.9 × 10^9^/L (7.0-28.1 × 10^9^/L), and CRP in patients had a median count of 106.9 mg/L (28.8-311.0 mg/L).

### Microbiologic characterization of the *K. pneumoniae* strains

Only 1 of 45 *K. pneumoniae* strains was ESBL-positive. All the isolates were resistant to ampicillin, but eleven were unsusceptible to piperacillin, two to cefuroxime, one to cefotaxime, one to ciprofloxacin, one to gentamicin, and one to cefepime (Table 2). All the isolates were susceptible to amikacin, amoxicillin-clavulanate, aztreonam, ceftazidime, cefoperazone-sulbactam, SMZ-TMP, imipenem, and meropenem, as shown in Table 2.

**Table 2:** **Microbiological characteristics of** ***K. pneumoniae*** **liver abscess**

**Microbiological characteristic**	**Value (n, %)**
Serotypes	
K1	31(68.9%)
K2	9(20%)
Non K1/K2	5(11.1%)
Virulence genes	
magA+	31(68.9%)
rmpA+	45(100.0%)
MLST	
ST23	26(57.8%)
Non-ST23	19(42.2%)
hvKP	13(28.9%)
cKP	32(71.1%)
ESBLs positive	1(2.2%)
**Antimicrobial susceptibility (S%)**	
Amikacin	100%
Amoxicillin-clavulanate	100%
Ampicillin	0%
Ampicillin-sulbactam	93.5%
Aztreonam	100%
Cefoxitin	95.7%
Cefuroxime	95.7%
Cefepime	97.8%
Cefotaxime	97.8%
Ceftazidime	100%
Ciprofloxacin	97.8%
Gentamicin ,CN10	97.8%
Piperacillin	76.1%
Piperacillin-tazobactam	97.8%
Cefoperazone-sulbactam	100%
Tetracycline	93.5%
Minocycline	93.5%
SMZ-TMP	100%
Imipenem	100%
Meropenem	100%

**Figure 1:**
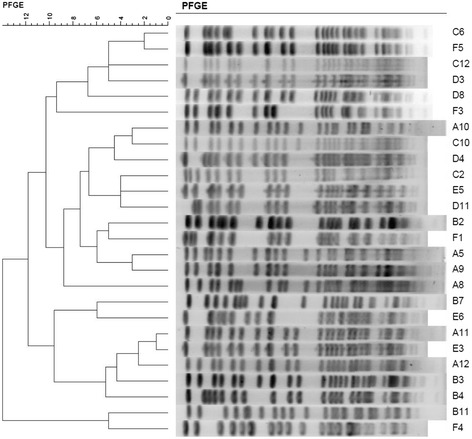
**PFGE analysis of the 26 ST23** ***K. pneumoniae*** **strains.**

MLST analysis revealed a total of 15 genotypes among the 45 *K. pneumoniae* strains. With respect to the sequence types (ST), more than half were ST23 (n =26, 57.8%), followed by ST65 and ST86 (both n = 3), ST375 (n = 2), and ST25, ST29, ST30, ST163, ST367, ST374, ST380, ST660, ST700, ST806, and ST1049 (n =1 for all), as shown in Additional file 1: Table S1. The PFGE results showed that the 26 ST23 *K. pneumoniae* strains had 12 separated pulsetypes, as shown in Figure 1.

Among 45 *K. pneumoniae* strains, K1 was the dominant serotype (31 strains, 68.9%), followed by K2 serotype (9 strains, 20%) and non K1/K2 serotype (5 strains, 11.1%). As shown in Additional file 1: Table S1, 31 serotype K1 KLA *K. pneumoniae* isolates mainly belonged to ST23-like (83.9%), while STs of these 9 serotype K2 isolates were ST65-like (5 strains), ST86-like (2 strains), ST23-like (1 strain), and ST374 (1 strain).

Virulence genes analysis showed 31 strains (68.9%) were *magA* gene positive and all 45 strains were *rmpA* gene positive, as shown in Table 2. According to the string test results, 13 hvKP isolates were obtained at the rate of 28.9%. All of the 13 hvKP strains belonged to K1/K2 serotype (7 K1 isolates and 6 K2 isolates).

### Comparison of microbiological characteristics between ST23 and non-ST23 K. pneumoniae isolates

As shown in Table 3, the prevalence of K1 serotype or *magA*-positive isolates in ST23 *K. pneumoniae* isolates was significantly higher than that in non-ST23 isolates (96.2% *vs* 31.6%, *P* < 0.001). Conversely, the prevalence of K2 serotype in ST23 *K. pneumoniae* isolates was significantly lower than that in non-ST23 isolates (0% *vs* 47.4%, *P* < 0.001).

**Table 3:** **Comparison of microbiological characteristics between ST23 and non-ST23** ***K. pneumoniae*** **isolates from KLA**

**Microbiological characteristic**	**ST23 (n = 26)**	**Non-ST23 (n = 19)**	***P*** **-value**
	**No. (%)**	**No. (%)**	
Serotypes -no. (%)			
K1	25(96.2%)	6(31.6%)	<0.001
K2	0(0.0%)	9(47.4%)	<0.001
Non K1/K2	1(3.8%)	4(21.1%)	0.146
Virulence genes -no. (%)			
magA+	25(96.2%)	6(31.6%)	<0.001
rmpA+	26(100.0%)	19(100%)	1
hvKP -no. (%)	6(23.1%)	7(36.8%)	0.341
cKP -no. (%)	20(76.9%)	12(63.2%)	0.341
ESBLs positive -no. (%)	1(3.8%)	0(0.0%)	0.387

### Comparison of microbiological characteristics between hvKP and cKP isolates from KLA

As shown in Table 4, the prevalence of K2 serotype in hvKP isolates was significantly higher than that in cKP isolates (46.2% *vs* 9.4%, *P* = 0.011). All of the 5 non-K1/K2 serotype isolates belonged to cKP, including 1 ST23 and 4 non-ST23 isolates. No significant difference was found between the *magA* positive rates in hvKP and cKP strains (53.8% *vs* 75%).

**Table 4:** **Comparison of microbiological characteristics between hvKP and cKP isolates from KLA**

**Microbiological characteristic**	**hvKP (n = 13)**	**cKP (n = 32)**	***P*** **-value**
	**No. (%)**	**No. (%)**	
Serotypes -no. (%)			
K1	7(53.8%)	24(75%)	0.286
K2	6(46.2%)	3(9.4%)	0.011
Non K1/K2	0(0.0%)	5(15.6%)	0.301
Virulence genes -no. (%)			
*magA*+	7(53.8%)	24(75%)	0.286
*rmpA*+	13(100%)	32(100%)	
ST23-no. (%)	6(46.2%)	20(62.5%)	0.341
Non-ST23 -no. (%)	7(53.8%)	12(37.5%)	0.341
ESBLs positive -no. (%)	1(7.7%)	0(0.0%)	0.219

### Comparison of clinical characteristics of KLA patients infected with different *K. pneumoniae* isolates

#### Comparison of clinical characteristics between KLA patients infected with magA-negative and -positive K. pneumoniae isolates

As shown in Table 5, there is a trend that more patients with biliary tract diseases in KLA patients infected with *magA*-negative *K. pneumoniae* isolates than those in patients with *magA*-positive *K. pneumoniae* isolates (57.1% *vs* 25.8%, *P* = 0.053). There were no significant differences of liver abscess sizes and complications between *magA*-positive and -negative *K. pneumoniae* isolates.

**Table 5:** **Comparison of clinical characteristics of KLA patients infected with different** ***K. pneumoniae*** **isolates**

**Clinical characteristic**	**ST23 and non-ST23**			**hvKP and cKP**			**magA-positive and -negative**		
	**ST23 (n = 26)**	**Non-ST23 (n = 19)**	**P**	**hvKP (n = 13)**	**cKP (n = 32)**	**P**	**magA positive (n = 31)**	**magA negative (n = 14)**	**P**
	**No. (%)**	**No. (%)**		**No. (%)**	**No. (%)**		**No. (%)**	**No. (%)**	
Male sex	17(65.4%)	11(57.9%)	0.757	8(61.5%)	18(56.3%)	1	17(54.8%)	9(64.3%)	0.746
Age	54.8 ± 11.9	52.3 ± 10.4	0.465	52.3 ± 8.7	54.3 ± 12.2	0.600	53.9 ± 12.5	53.2 ± 8.2	0.844
**Coexisting condition**									
Diabetes mellitus	13(50.0%)	9(47.4%)	1	4(30.8%)	18(56.3%)	0.189	16(50%)	6(42.9%)	0.749
Biliary tract diseases	8(30.8%)	8(17.8%)	0.534	4(30.8%)	12(37.5%)	0.743	8(25.8%)	8(57.1%)	0.053
Abdominal surgery	6(23.1%)	4(21.1%)	1	3(23.1%)	7(21.9%)	1	7(22.6%)	3(21.4%)	1
**No underlying diseases**	3(11.5%)	2(5.3%)	1	3(23.1%)	2(6.3%)	0.136	3(9.7%)	2(14.3%)	0.639
**Current drinker**	10(38.5%)	4(21.1%)	0.330	2(15.4%)	12(37.5%)	0.178	11(35.5%)	3(21.4%)	0.492
Abscess size (diameter, cm)	7.7 ± 2.4	7.4 ± 2.8	0.691	8.8 ± 2.4	7.1 ± 2.5	0.057	7.5 ± 2.3	7.9 ± 3.0	0.605
**Complications**									
Metastatic infections	10(38.5%)	4(21.1%)	0.330	5(38.5%)	9(28.1%)	0.486	11(35.5%)	3(21.4%)	0.492
Spontaneous bacterial peritonitis	3(11.5%)	0(0.0%)	1	0(0.0%)	3(9.4%)	0.546	3(9.7%)	0(0.0%)	0.541
Pneumonia	6(23.1%)	4(21.1%)	1	3(23.1%)	7(21.9%)	1	7(22.6%)	3(21.4%)	1
Endophthalmitis	1(3.8%)	0(0.0%)	1	0(0.0%)	1(3.1%)	1	1(3.2%)	0(0.0%)	1
Septic shock	3(11.5%)	0(0.0%)	0.258	2(15.4%)	1(3.1%)	0.196	3(9.7%)	0(0.0%)	0.541

#### Comparison of clinical characteristics between KLA patients infected with hvKP and cKP isolates

More patients infected with cKP tend to have a history of diabetes mellitus or drinking than that of hvKP patients (56.3% *vs* 30.8% and 37.5% *vs* 15.4% respectively, *P* = 0.189 and 0.178, respectively). There were 3 hvKP (3/13, 23.1%) patients and 2 cKP (2/32, 6.3%) without any underlying diseases. Liver abscess sizes in patients infected with hvKP (8.8 ± 2.4 cm) were tend to be larger than those in patients infected with cKP (7.1 ± 2.5 cm) (*P* = 0.057) (Table 5).

#### Comparison of clinical characteristics between KLA patients infected with ST23 and non-ST23 K. pneumoniae isolates

Among 26 patients infected with ST23 *K. pneumoniae* strains, 21 patients (80.8%) had solitary abscess and 5 patients (19.2%) had multiple abscesses. However, all of 19 patients (100%) infected with non-ST23 *K. pneumoniae* strains had solitary abscess (P = 0.043 *vs* ST23 strains). There were no significant differences of liver abscess sizes and complications between ST23 and non-ST23 *K. pneumoniae* isolates (Table 5).

## Discussion

Pyogenic liver abscess is a potentially life-threatening disease. Although *Escherichia coli* was the most common pathogen in liver abscess before the 1980s, *K. pneumoniae* had become the dominant pathogen for liver abscess during the past two decades. In Taiwan, over 80% of bacterial liver abscesses were caused by *K. pneumoniae*. In Singapore and South Korea, the prevalence of KLA was similar. In our medical center, approximate 81.7% of pyogenic liver abscess were caused by *K. pneumoniae*. Male gender, patients with diabetes mellitus and biliary disease were more likely to have KLA. Most patients had a single abscess on the right hepatic lobe. The low mortality rate has been similarly observed in recent reports on liver abscess [20,21].

Although liver abscesses caused by extended spectrum β-lactamase (ESBL)-producing *K. pneumoniae* have been reported, it is a rare occurrence [21]. In our study, only 1 *K. pneumoniae* strain produced ESBL, which was isolated from a KLA patient who had an accident injury one month ago. As previous reported, *K. pneumoniae* isolates from KLA were susceptible to almost all kinds of antimicrobial agents such as third generation cephalosporins, β-lactamase inhibitor compounds, and carbapenems [2,22].

The K1 *K. pneumoniae* isolates contributed to 68.9% of KLA in this study, which was higher than previous reports from mainland China (43% and 39.2%) [15,20,23]. The prevalence of K1 isolates was significantly higher than K2 in this study (68.9% *vs* 20%). Our MLST data of the *K. pneumoniae* isolates from KLA patients revealed that ST23 was predominant sequence type with a rate of 57.8%, which was higher than that in the recent report from North China (37.2%) [15]. This indicated that serotypes and MLST of KLA *K. pneumoniae* maybe different among various regions in China. Nationwide study would be necessary to realize the national molecular epidemiology of KLA *K. pneumoniae* in China. In our study, serotype K1 KLA *K. pneumonia* isolates that mainly belong to ST23, while ST65-like and ST86-like are the two major MLST types among serotype K2 isolates. These results were similar with Siu-LK et al’s recent report from Asia [14].

PFGE results demonstrated that there was no clonal dissemination among the 26 ST23 *K. pneumoniae* isolates. Most of ST23 *K. pneumoniae* isolates (96.2%) belonged to K1 serotype with *magA* positive. As previously reported, ST23 was the most prevalent sequence type among serotype K1 *K. pneumoniae* isolates from both liver abscess and stool samples in the Asia Pacific region [2,24-26]. It implies that liver abscess might develop after leakage of *K. pneumoniae* from a patient’s bowel into their liver via the portal circulation [25-27].

*magA* has been described as the causative gene for *K. pneumoniae* liver abscess and septic metastatic complications [7]. The enzyme encoded by *magA*, also named *wzy* in accordance with the bacterial polysaccharide gene nomenclature scheme, functions as a polymerase involved in capsule synthesis, and this function is restricted to the capsular gene cluster of serotype K1 only [10,28]. In previous study, *magA* has been reported in 98.1% and 83.3% of *K. pneumoniae* strains isolated from patients with liver abscess and was significantly more prevalent than the bacteremic strains [17,28,29]. However, our study showed that *magA* was detected in 68.9% of *K. pneumoniae* isolates from KLA patients, and 96.2% in ST23 *K. pneumoniae* isolates from KLA patients. Therefore, almost all ST23 *K. pneumoniae* isolates from KLA patients was *magA*-positive and K1 serotype, but not all of the *magA*-positive and K1 serotype *K. pneumoniae* isolates from KLA patients belonged to ST23.

*rmpA* has been confirmed to regulate capsular polysaccharide synthesis and was proposed as a virulent factor in addition to *magA* and capsular serotypes K1/K2 [8,30]. Recently, *rmpA*-associated hypermucoviscosity phenotype has also been reported to play an important role in invasive purulent diseases caused by *K. pneumoniae. K. pneumoniae* serotypes K1 and K2 isolated from patients with liver abscess usually carrying hypermucoviscosity [2,5]. Our study showed that all *K. pneumoniae* strains that cause liver abscesses were *rmpA*-positive. However, our string test results revealed that only 28.9% *K. pneumoniae* isolates from KLA showed hypermucoviscosity. The hvKP rate was significantly lower than that reported by previous studies (more than 85%) and also the recent report from mainland China (70.6%) [5,8,15]. We had repeated the string test for three times and received the same results. It seemed that string test might not be a reliable method to identify *K. pneumoniae* with the potential to cause KLA. All of the 13 *K. pneumoniae* strains with hypermucoviscosity (hvKP) belonged to K1/K2 serotype. The prevalence of cKP in *K. pneumoniae* isolates from diabetes mellitus and drinker patients were higher than that of hvKP. It was consistence with that hvKP was more liable to cause KLA in healthy hosts than cKP [5]. However, there was no significant difference of complications and prognosis between patients infected with hvKP and cKP, ST23 and non-ST23, or *magA*-positive and -negative strains.

Our study also has several limitations. First, any potential for selection bias in terms of those PLAs aspirated or those patients presenting to this particular institute. Second, that there is no denominator data here; neither in terms of number of PLAs in total, not of number of *K. pneumoniae* invasive isolates (i.e. positive blood cultures) which did not result in PLA. Third, although there was no significant difference of complications and prognosis among patients infected with different *K. p*neum*oniae* isolates, the sample size of our study was relative small. In order to determine the predictor of metastatic infection and prognosis of KLA*,* more KLA cases should be collected for further study.

## Conclusions

In summary, K1 serotype and ST23 were the predominant serotype and sequence type of KLA *K. pneumoniae* in our study. The hvKP rate was significantly lower than that in previous reports. There was no significant association between the microbiological characteristics including serotypes, *magA* and *rmpA* genotypes, and STs with the metastatic infection and prognosis of KLA in this study. Further studies with larger sample are needed in the future.

## Additional file

Additional file 1: Table S1. MLST and serotype analysis in *K. pneumoniae* isolates from liver abscess.
