# Supplementary material for: Clinical and microbiological characteristics of Klebsiella pneumoniae liver abscess in East China
Source: BMC Infect Dis. 2015 Mar 27;15:161. doi: 10.1186/s12879-015-0899-7 (PMC4381403; doi:10.1186/s12879-015-0899-7)
Supplement: Additional file 1: Table S1. — MLST and serotype analysis in K. pneumoniae isolates from liver abscess. [file 12879_2015_899_MOESM1_ESM.docx]

Additional file 1

Supplementary Table.S1 MLST and serotype analysis in *K. pneumoniae* isolates from liver abscess

| **ST**  **(no. of isolates)** | **ST group** | **serotype**  **(no. of isolates)** | **Allelic profiles** | | | | | | |
| --- | --- | --- | --- | --- | --- | --- | --- | --- | --- |
|  |  |  | **gapA** | **infB** | **mdh** | **pgi** | **phoE** | **rpoB** | **tonB** |
| ST23(26) | ST23-like group | K1(25);Non K1/K2(1) | 2 | 1 | 1 | 1 | 9 | 4 | 12 |
| ST25(1) | ST23-like group | Non K1/K2 | 2 | 1 | 1 | 1 | 10 | 4 | 13 |
| ST163(1) | ST23-like group | K1 | 2 | 1 | 1 | 1 | 9 | 1 | 12 |
| ST380(1) | ST23-like group | K2 | 2 | 1 | 1 | 1 | 1 | 4 | 19 |
| ST65(3) | ST65-like group | K2(3) | 2 | 1 | 2 | 1 | 10 | 4 | 13 |
| ST375(2) | ST65-like group | K2(2) | 43 | 1 | 2 | 1 | 10 | 4 | 13 |
| ST86(3) | ST86-like group | K2(2); K1(1) | 9 | 4 | 2 | 1 | 1 | 1 | 27 |
| ST29(1) | separated | Non K1/K2 | 2 | 3 | 2 | 2 | 6 | 4 | 4 |
| ST30(1) | separated | Non K1/K2 | 5 | 3 | 1 | 1 | 9 | 4 | 26 |
| ST367(1) | separated | K1 | 18 | 15 | 26 | 16 | 11 | 13 | 106 |
| ST374(1) | separated | K2 | 2 | 3 | 58 | 37 | 10 | 27 | 9 |
| ST660(1) | separated | Non K1/K2 | 2 | 1 | 2 | 1 | 4 | 1 | 25 |
| ST700(1) | separated | K1 | 10 | 1 | 17 | 37 | 12 | 1 | 9 |
| ST806(1) | separated | K1 | 2 | 1 | 1 | 1 | 89 | 1 | 44 |
| ST1049(1) | separated | K1 | 2 | 3 | 4 | 97 | 12 | 1 | 39 |
